# Combination therapy with ampicillin and azithromycin improved outcomes in a mouse model of group B streptococcal sepsis

**DOI:** 10.1371/journal.pone.0182023

**Published:** 2017-07-31

**Authors:** Kirtikumar Upadhyay, Basu Hiregoudar, Elizabeth Meals, Boyce Keith English, Ajay J. Talati

**Affiliations:** 1 Children’s Foundation Research Center at Le Bonheur Children’s Hospital; University of Tennessee Health Science Center, Memphis, TN, United States of America; 2 Division of Neonatal-Perinatal Medicine; University of Tennessee Health Science Center, Memphis, TN, United States of America; 3 Departments of Pediatrics; University of Tennessee Health Science Center, Memphis, TN, United States of America; 4 Cheyenne Regional Medical Center, Cheyenne, WY, United States of America; 5 Department of Pediatrics and Human Development, College of Human Medicine, Michigan State University, Lansing, MI, United States of America; 6 Department of Obstetrics-Gynecology; University of Tennessee Health Science Center, Memphis, TN, United States of America; Kaohsiung Medical University, TAIWAN

## Abstract

**Background:**

Evidence suggests that β-lactam monotherapy of streptococcal infections may incite stronger inflammation and is inferior to combination therapy with macrolides. We hypothesized that use of macrolides alone or in combination with a β-lactam for group B streptococcal (GBS) sepsis would improve outcomes by reducing inflammation.

**Methods:**

TNF-α was measured from supernatants of RAW 264.7 cells stimulated with GBS isolates, in presence of four treatment regimens: ampicillin alone, azithromycin alone, or combination of azithromycin plus ampicillin. Mouse model of GBS sepsis was developed and treated with same four regimens. Clinical sepsis scores were monitored; serum cytokines (TNF-α, IL-6, IL-10) and chemokines (MIP-1α) were measured at the end.

**Results:**

GBS isolates exposed to azithromycin or combination (compared to ampicillin alone) stimulated less TNF production *in vitro*. In the murine sepsis model, mortality was lower along with decreased sepsis scores in mice treated with combination therapy. Mean serum IL-6 was lower in mice treated with azithromycin alone (66±52 pg/ml) or combination of ampicillin plus azithromycin (52±22 pg/ml) compared to ampicillin alone (260±160 pg/ml) (p<0.005).

**Conclusions:**

Combination therapy of ampicillin+azithromycin improved outcomes in a murine GBS sepsis model; this therapeutic approach deserves additional study.

## Introduction

Group B streptococci (GBS) have long been recognized as a leading cause of infections in neonates, young infants, and pregnant women [1, 2]. Despite increased clinical awareness, prompt diagnosis and aggressive therapy, GBS is still the most common cause of sepsis in neonates [3]. Although the case fatality rate has declined significantly with increasing intensive care, approximately 50% of infants who survive from GBS meningitis have permanent neurological damage [4].

Since Austrian and Gold [5] first demonstrated the efficacy of penicillin in the treatment of adults with streptococcal infections >50 years ago, penicillin or other β-lactam agents have been considered to be the treatment of choice for most patients. The Infectious Diseases Society of America (IDSA) specifically recommends β-lactam agents as the first line of therapy for streptococcal infections [6]. Beta lactam antibiotics work by inhibiting peptide bond formation in the bacterial cell wall, which in turn leads to bacterial lysis. [7]. Host defense against GBS infection in infants primarily relies on the innate immune system to initiate a response that is characterized by local and systemic production of anti- and pro-inflammatory signaling intermediates, cytokines (such as TNF-α, IFN-γ, IL-1β, IL-6, IL-12, and IL-18), and nitric oxide [8]. The resultant innate immune response limits the early proliferation and spread of GBS [9–11]. The general goal of treatment has been to eliminate pathogens as rapidly as possible, so bactericidal agents have been preferred. These bactericidal antibiotics cause rapid release of bacterial cell wall and other components, which may result in an augmented and potentially harmful systemic inflammatory response [12,13]. These exaggerated proinflammatory responses in the context of overwhelming GBS infection may contribute to many of the manifestations of GBS diseases including high morbidity and mortality.

In the past few years, studies of experimental murine models of pneumococcal pneumonia by Karlstrom and colleagues [14] and clinical studies by Mufson and Stanek [15], Waterer *et al*. [16], Martinez *et al*. [17] and Baddour *et al*. [18] have all identified significant mortality reductions in patients with bacteremic pneumococcal pneumonia who received combination antibiotic therapy (beta lactam with macrolide) in comparison with patients who received monotherapy with beta lactam antibiotics. Macrolide group of antibiotics (e.g. azithromycin) are bacteriostatic and inhibit bacterial protein synthesis by reversible binding to the P site of the 50S subunit of the bacterial ribosome. As a consequence of their primary ribosomal-targeted mechanism of antimicrobial action, they inhibit the production of proinflammatory microbial toxins and other virulence factors [19,20].

Little attention has been paid to potential role of macrolide group of antibiotics in modulating the host response to GBS infection. To date, no data exist on use of macrolide group of antibiotics for GBS infections. The ideal antimicrobial agent should effectively eradicate the infection while leading to a less pronounced inflammatory response, which may lead to reduced morbidities and mortality.

We hypothesized that treatment of GBS infected mice with combination antibiotics of azithromycin and ampicillin will be superior and would result in lower concentration of inflammatory cytokines *in vitro* and *in vivo*, than treatment with ampicillin alone.

## Results

### Macrophage experiments

**Fig 1:**
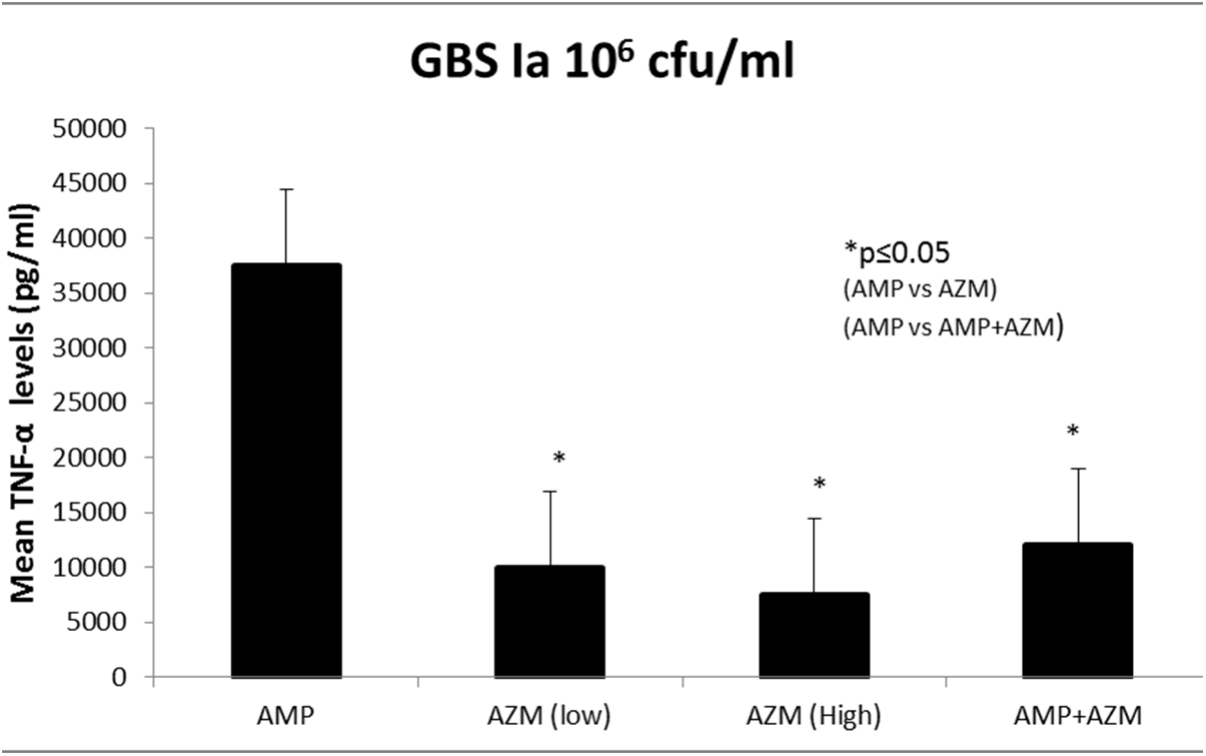
GBS Ia mediated murine macrophages secreted lower TNF-α with AMP+AZM compared to AMP alone. Mean TNF production (pg/mL) ± SD by RAW 264.7 cells in response to stimulation with 10^6^ cfu/mL of GBS Ia, when treated with ampicillin alone at(20mg/L), azithromycin (AZM) alone at (5mg/L and 20mg/L) and combination antibiotics ampicillin (20mg/L) and azithromycin (20mg/L) is shown. * = p ≤0.01.

We first compared murine macrophage RAW 264.7 cells’ TNF-α secretion in response to stimulation with an azithromycin susceptible GBS 1a isolate in the presence of ampicillin, azithromycin, or ampicillin + azithromycin. Fig 1 depicts mean TNF-α secretion (n = 7) using GBS (10^6^ cfu/mL). Analysis of variance (ANOVA) on these levels yielded significant differences and post hoc tests showed that; exposure of GBS to azithromycin alone at (5mg/L and 20 mg/L) led to significantly less TNF-α secretion compared with exposure to ampicillin (26% less than ampicillin, *p* = 0.008). Similarly, exposure of GBS to combinations of ampicillin (20mg/L) plus azithromycin (20mg/L) led to significantly less TNF-α secretion (36% less than ampicillin alone, p = 0.01).

**Fig 2:**
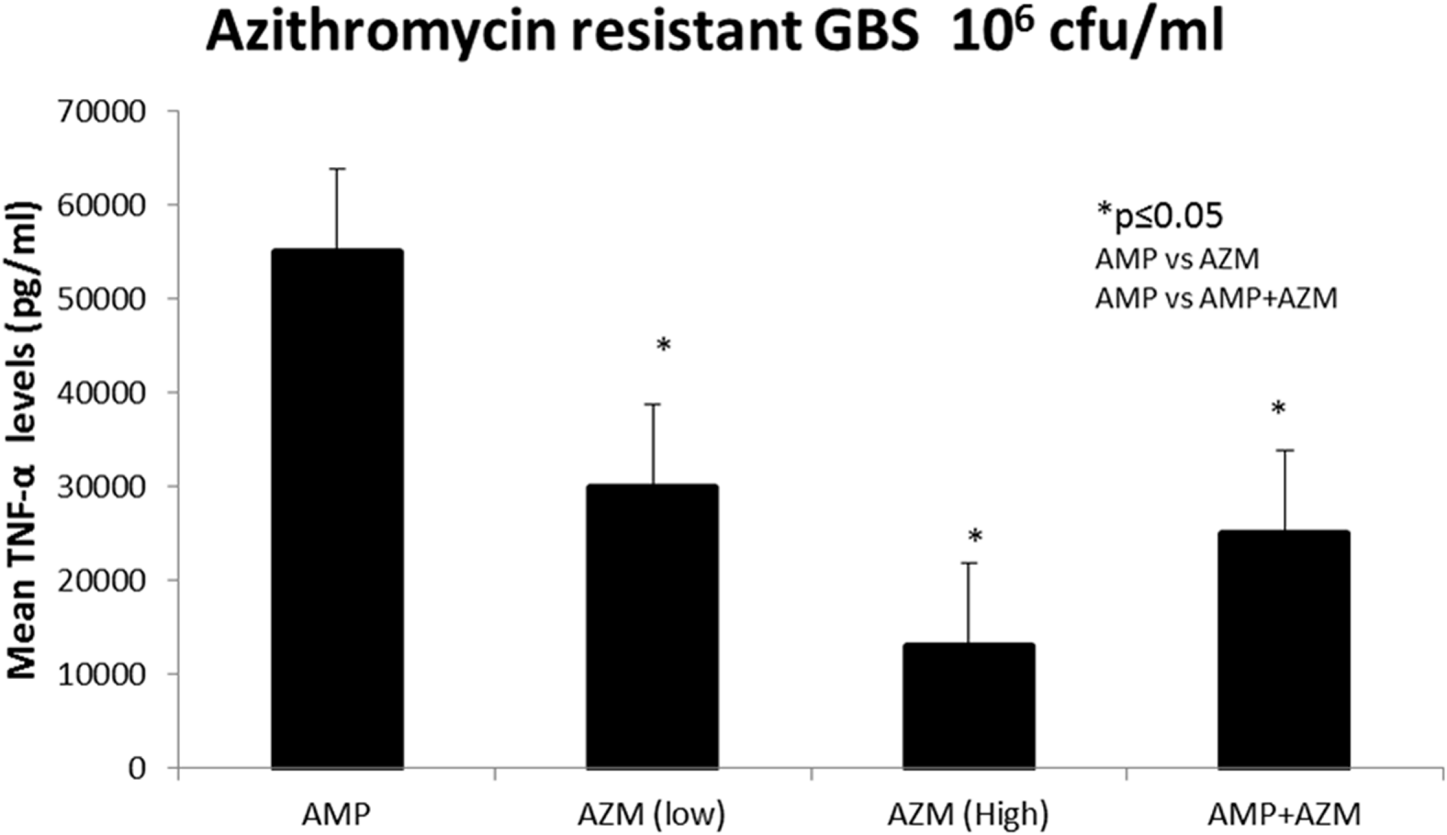
Azithromycin resistant GBS mediated murine macrophages secreted lower TNF-α with AMP+AZM compared to AMP alone. Mean± SD TNF production (pg/mL) by RAW 264.7 cells in response to stimulation with 10 ^6^ cfu/mL of azithromycin resistant GBS, when treated with ampicillin alone at (20mg/L), azithromycin alone at (5mg/L and 20mg/L) and combination antibiotics ampicillin (20mg/L) and azithromycin (20mg/L) is shown. * = p ≤0.01.

We then compared the effects of ampicillin, azithromycin, or ampicillin+azithromycin on murine macrophage TNF-α response to an azithromycin resistant strain of GBS. Fig 2 depicts mean TNF-α secretion (n = 6) using azithromycin resistant GBS at 10^6^ cfu/mL. As shown in Fig 2, when compared with exposure to beta-lactam antibiotics, exposure of azithromycin resistant- GBS to azithromycin alone at (5mg/L and 20 mg/L) led to significantly less TNF-α secretion (54% less than ampicillin alone, *p* = 0.001). Similarly, exposure of azithromycin resistant-GBS to combinations antibiotics ampicillin (20mg/L) and azithromycin (20mg/L) led to significantly less TNF-α secretion (44% less than ampicillin alone, *p* = 0.0002).

Results from experiments using GBS isolate at 10^7^ cfu/mL were similar (n = 7) (not shown). Stimulation of RAW 264.7 cells with antibiotics alone did not produce any cytokines (negative control). Furthermore, RAW 264.7 cells when stimulated with heat killed GBS 10^6^ CFU/ml released 2131 (±187) pg/ml of TNF-α (positive control). Cell viability assay with trypan blue exclusion method was performed for experimental conditions with RAW cells and GBS. This has also been previously reported from our laboratory [21]. Experiments with GBS type Ia without antibiotics (positive control) led to rapid growth of bacteria and fulminant cellular necrosis leading to poor cell viability.

### Murine GBS sepsis model experiments

**Fig 3:**
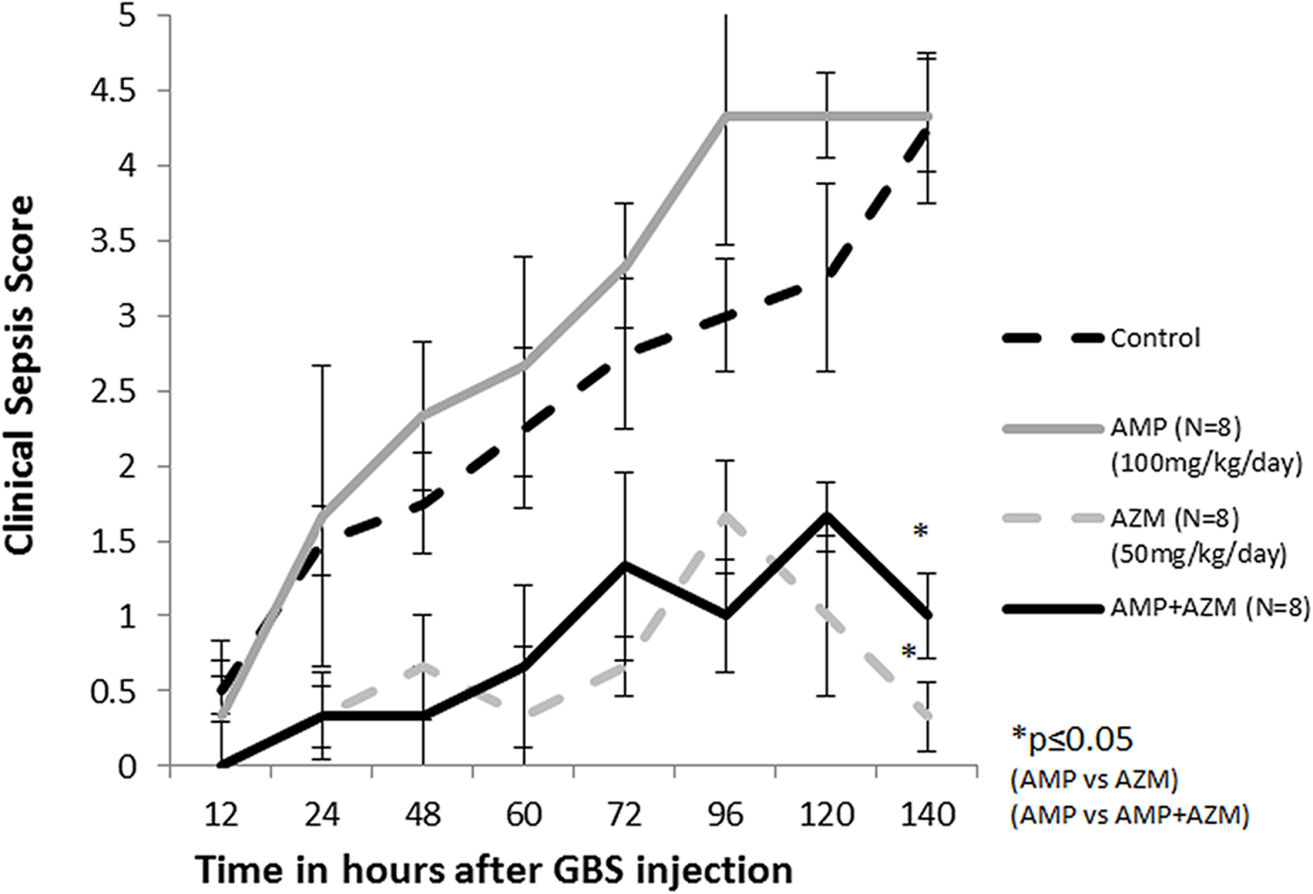
Clinical sepsis score in GBS infected mice treated with antibiotics. Mean clinical sepsis score of GBS infected mice in each group was calculated and plotted as shown. * = p ≤0.01.

**Fig 4:**
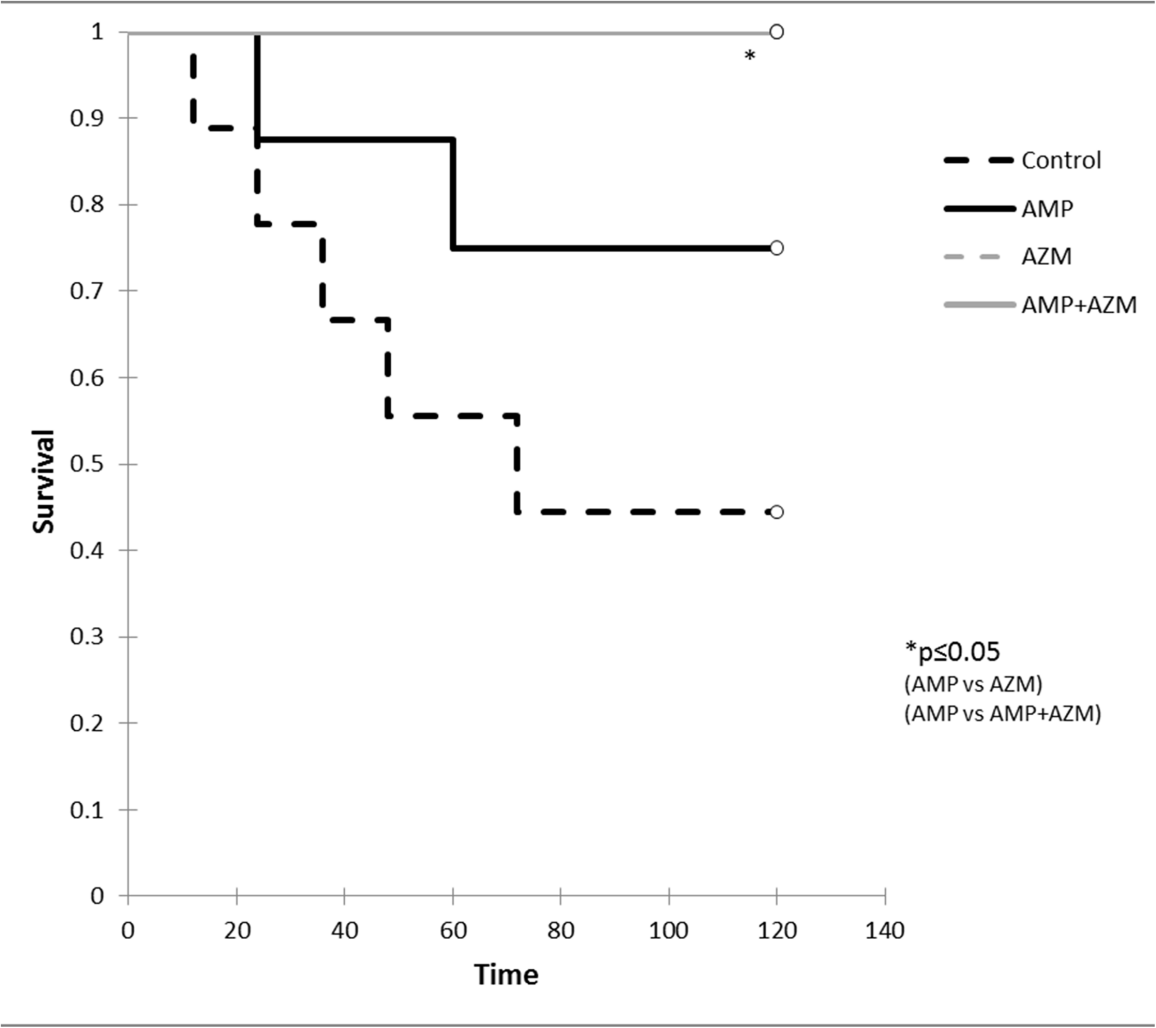
Survival analysis of GBS infected mice treated with antibiotics. Kaplan- Meier survival graph shows that mortality was higher in GBS infected mice treated with ampicillin alone (AMP) compared to azithromycin alone (AZM) or in combination with ampicillin and azithromycin (AMP+AZM).

The clinical sepsis score of GBS (10^8^ cfu/ml) infected mice treated with azithromycin alone or in combination with ampicillin remained significantly lower compared to that of mice treated with ampicillin alone and control (no antibiotics) group. Fig 3 represents the mean clinical sepsis score (n = 9 for control group, n = 8 for each treatment groups) at different time points during experiment. As seen in Fig 3, the mean clinical sepsis score of GBS infected mice treated with ampicillin alone was significantly higher compared to mean clinical sepsis score of GBS infected mice treated with combination of ampicillin and azithromycin (p = 0.002) (Fig 3). The case fatality of mice was lower in azithromycin alone (0/8) and in combination with ampicillin (0/8) compared to ampicillin alone (2/8) and control group (5/9). (Fig 4)

**Fig 5:**
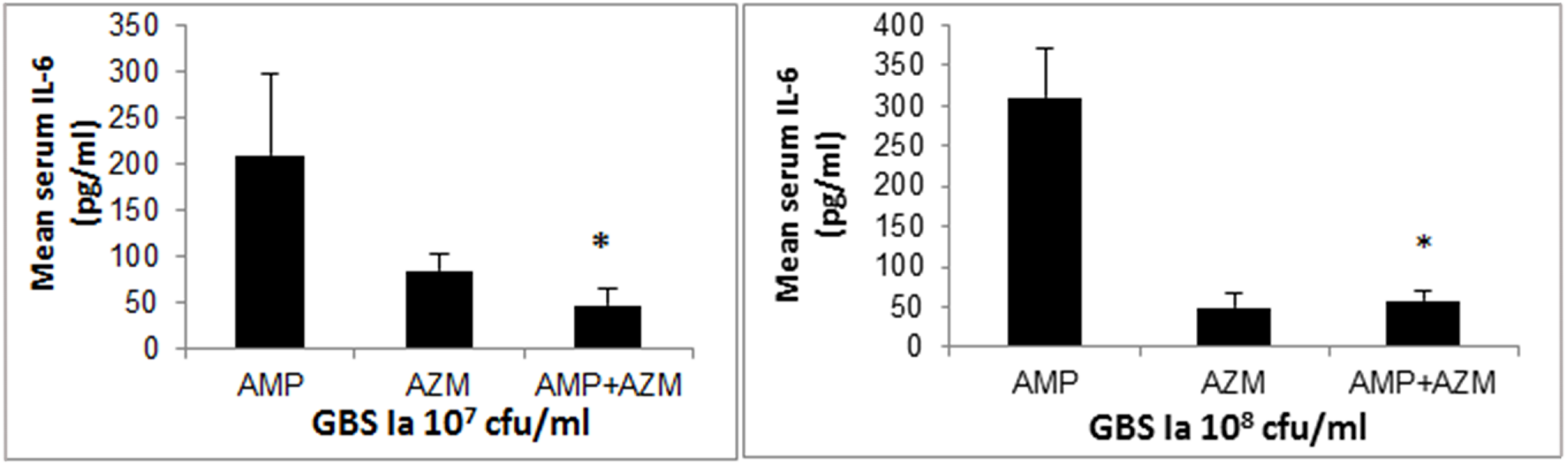
**5A and 5B: IL-6 levels in GBS infected mice.** Mean serum IL-6 levels were measured and shown among GBS infected mice treated with antibiotics. Fig 5A represent GBS inoculum 10^7^ cfu/ml and Fig 5B represent GBS inoculum 10^8^ cfu/ml. * = p<0.01.

Blood samples were collected immediately after death of the mice (mean age 96 hours). As seen in Fig 5A and 5B; mean serum IL-6 levels were higher in GBS infected mice treated with ampicillin alone compared to combination of ampicillin and azithromycin (p = 0.0037).

Blood cultures of untreated mice showed several colonies of GBS on a blood agar plate, while no bacterial colony was found from blood cultured from mice receiving ampicillin alone or in combination with azithromycin. Very little TNF-α, IL-10 and MIP-1α were detected in serum and no difference was found between the groups.

**Fig 6:**
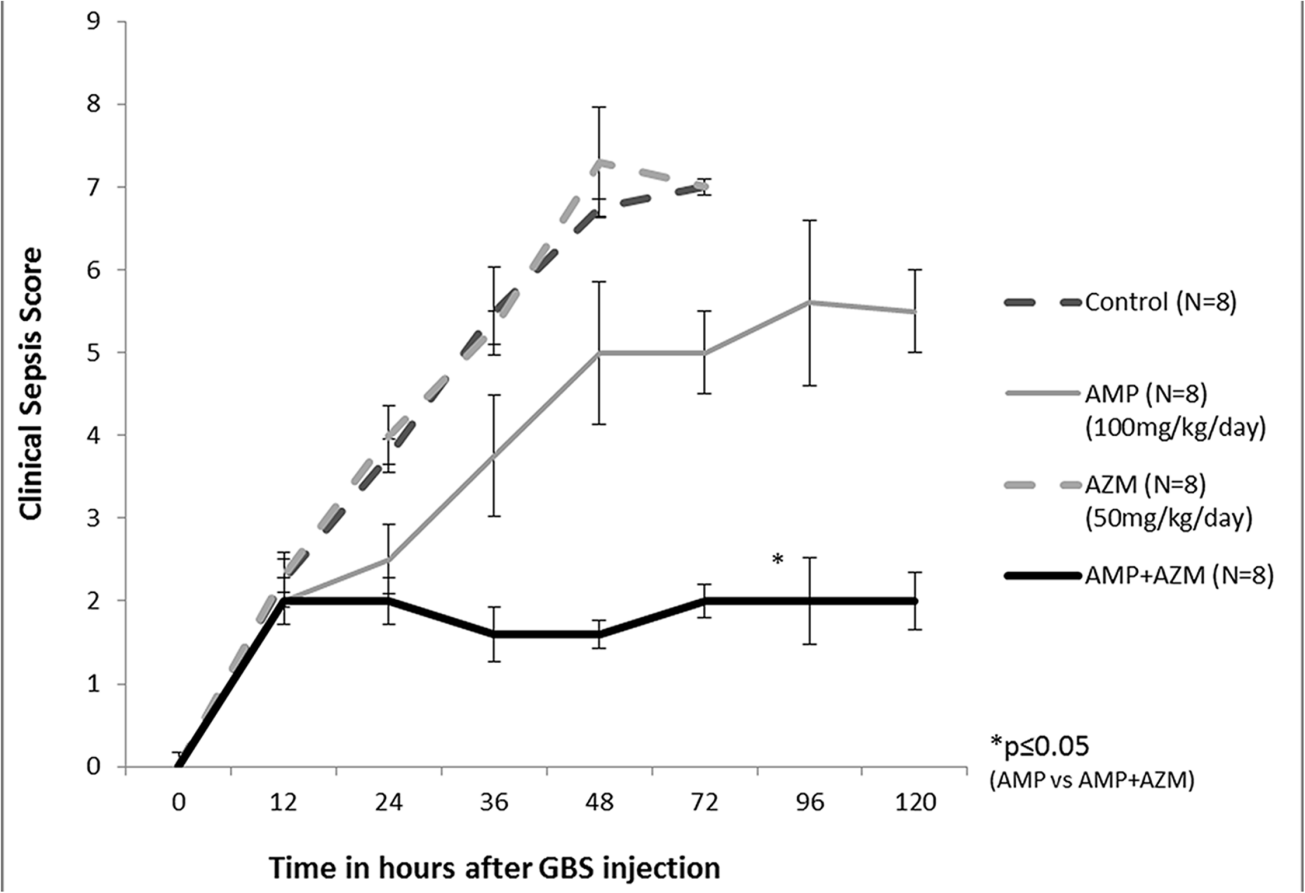
Clinical sepsis score in AZM resistant GBS infected mice treated with antibiotics. Mean clinical sepsis score of GBS infected mice in each group was calculated and plotted as shown. * = p ≤0.01.

**Fig 7:**
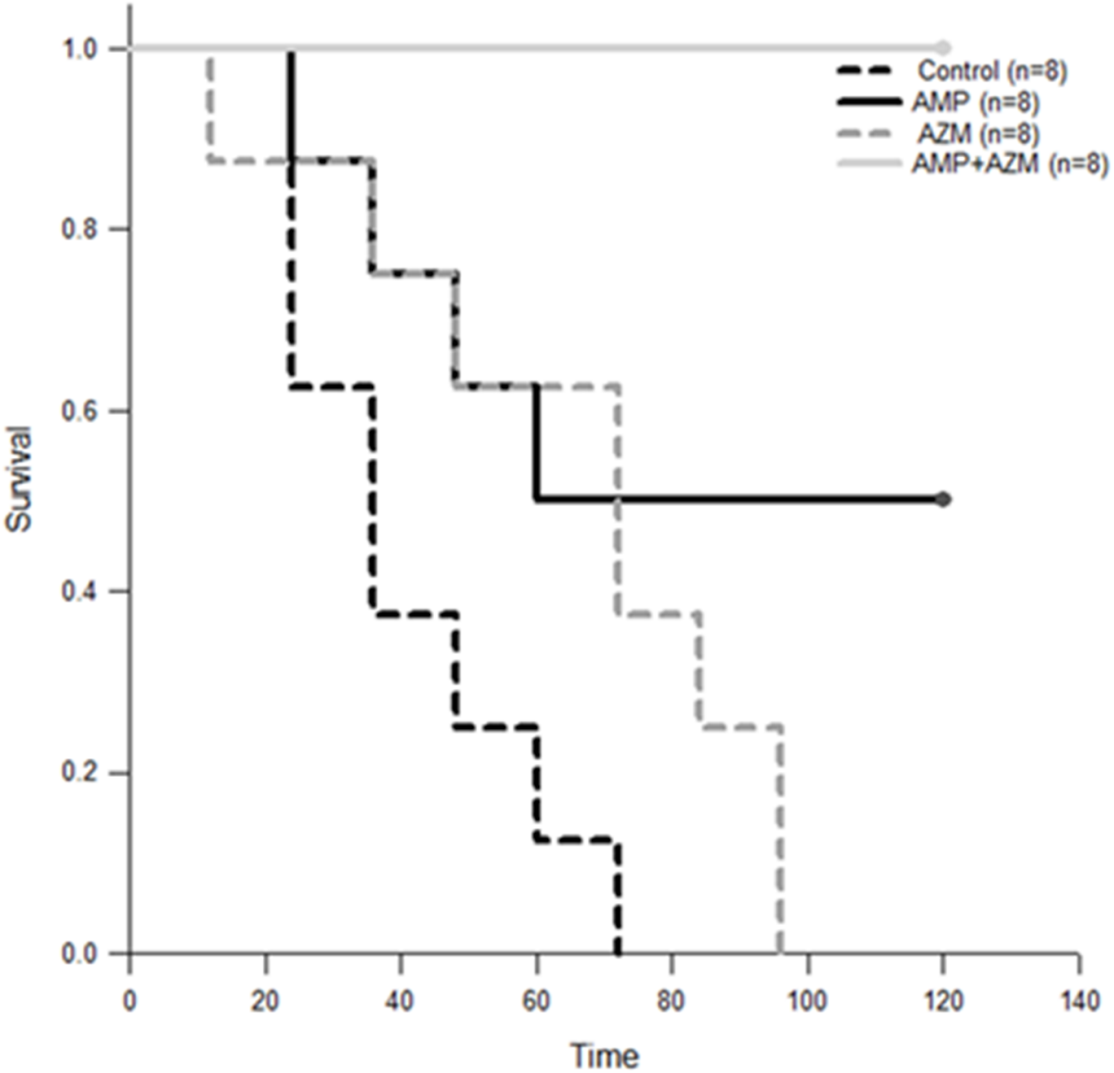
Survival analysis of AZM resistant GBS infected mice treated with antibitocs. Kaplan- Meier survival graph shows that mortality was higher in azithromycin resistant GBS infected mice treated with ampicillin alone (AMP) compared to combination with ampicillin and azithromycin (AMP+AZM).

**Fig 8:**
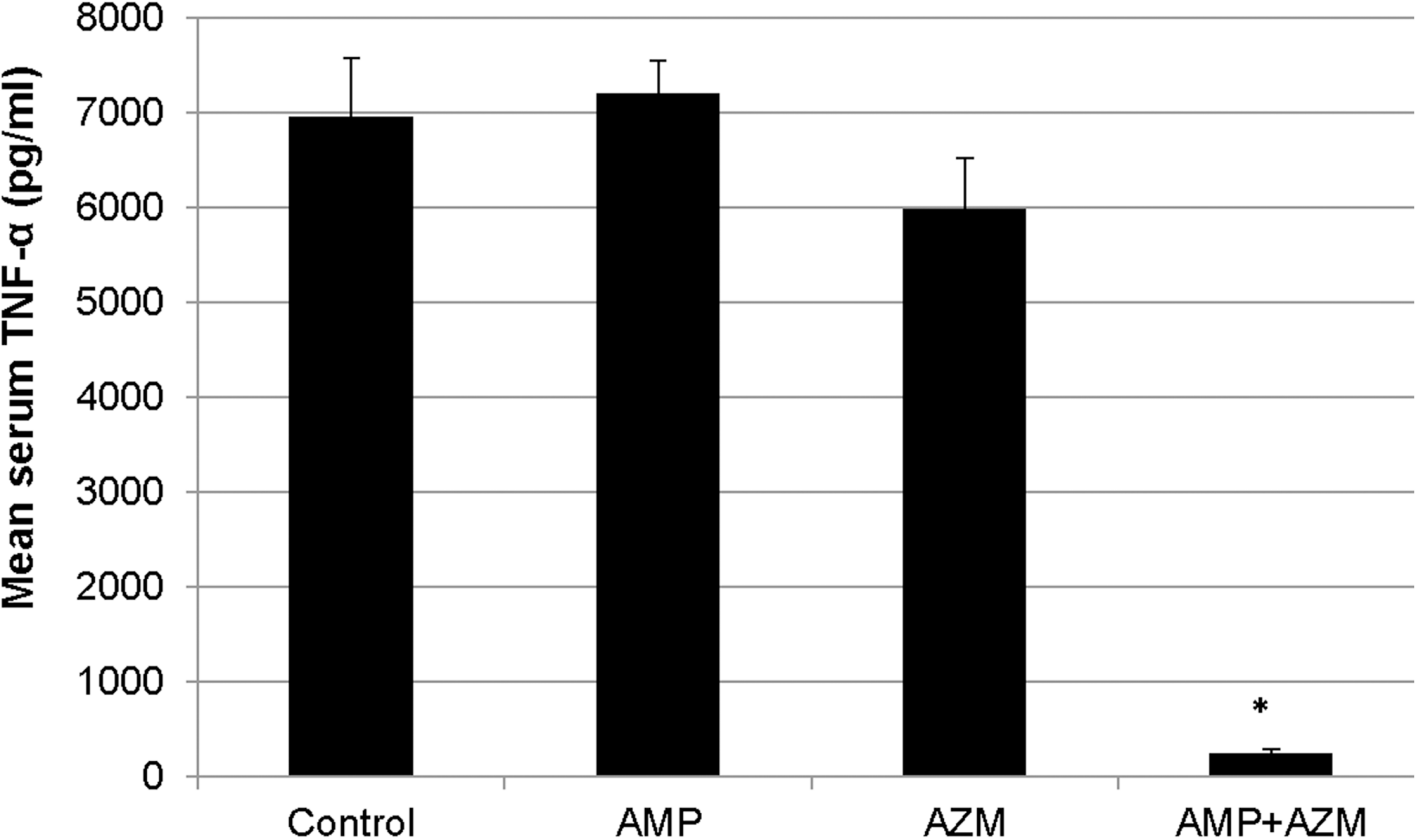
TNF-α levels in GBS infected mice treated with antibiotics. Mean serum TNF-α levels were measured and shown among GBS infected mice treated with antibiotics. * = p<0.01.

Similarly, the mean clinical sepsis score of azithromycin resistant-GBS infected mice (n = 32, 8 mice in each group) treated with ampicillin alone was significantly higher compared to mean clinical sepsis score of azithromycin resistant-GBS infected mice treated with combination of ampicillin+azithromycin (p = 0.003) (Fig 6). Mortality was lower in mice treated with ampicillin+azithromycin (0/8) compared to ampicillin alone (4/8) in experiment using azithromycin resistant GBS, however, all the mice in control group and azithromycin alone treatment group died (8/8) (Fig 7). From blood samples collected at death of mice (mean age 72 hours); we found that serum TNF-α levels were significantly higher in GBS infected mice treated with ampicillin alone compared to combination of ampicillin and azithromycin (p = 0.004) (Fig 8).

## Discussion

In our study, we found that GBS exposed to azithromycin (macrolide antibiotic) singly or in combination with ampicillin (*β*-lactam antibiotic) triggered less macrophage TNF secretion *in vitro* and induced less serum IL-6/TNF accumulation *in vivo* compared to ampicillin alone. More importantly, GBS infected mice treated with azithromycin alone or in combination with ampicillin had better survival and less severe sepsis scores.

Significant mortality rates are associated with GBS infection, despite the availability of effective and potent antibiotics for treatment. Even with appropriate therapy, mortality of severe GBS sepsis is as high as 20%–50% [22, 23]. Case fatality rates of invasive GBS sepsis, especially in preterm infants, have remained high in spite of intrapartum prophylaxis with penicillin [24, 25]. This may stem from the robust inflammatory response that occurs in response to severe infections. Use of β-lactam agents, such as ampicillin, may exacerbate the problem; these agents act by (1) lysing the bacteria and (2) releasing proinflammatory substances, such as cell-wall components, cytotoxins, and bacterial DNA, which are recognized by the innate immune system and which trigger the inflammatory response [26]. In support of this hypothesis, in our experiments, 25% mice infected with GBS type Ia, did not survive when treated with ampicillin, despite effective killing and clearance of bacteria. We extended this finding by using a more virulent strain of azithromycin resistant GBS. Use of only ampicillin therapy resulted in a case fatality rate of 50% in GBS infected mice. In these mice, treatment was associated with (1) increase in levels of proinflammatory cytokines, and (2) observation of more severe clinical sepsis score, despite rapid and complete clearance of bacteria. Combination of azithromycin and ampicillin improved survival to 100% and reduced inflammation in mice infected with either strain of GBS in our studies.

Similar approaches to therapy have previously been considered for severe bacterial infections. It is now well appreciated that the anti-inflammatory effects of steroids lead to improved outcomes in children with *Haemophilus influenzae* meningitis [27] or adults with pneumococcal meningitis [28]. Our findings are similar to those reported in both clinical trials and experimental models of pneumococcal pneumonia [29]. Although we used relatively high concentrations of GBS (10^6^ and 10^7^ cfu/mL) in our study, it corresponds to bacterial concentrations documented in some patients with serious infections such as meningitis [30]. In a rabbit model of pneumococcal meningitis, Nau et al. [31] demonstrated that treatment with a non-lytic antibiotic, such as rifampin, results in improved outcomes. Limited data from these investigators suggest that the benefits of rifampin therapy in this model are preserved when used in combination with ceftriaxone [31]. Smith et al [29] found in their study of pneumococcal pneumonia that treatment of pneumonia with protein synthesis inhibitor antibiotics, either alone or in combination with a *β*-lactam, may result in better outcomes by decreasing the inflammatory response engendered by lysis of the bacteria. In clinical trials, the reported effects of adding macrolides for therapy of pneumococcal pneumonia have been inconsistent. Retrospective studies [17] and a prospective, multicenter trial [18] have concluded that the addition of a macrolide to a β-lactam results in a significant reduction in mortality (compared with β-lactam therapy alone) in adults with bacteremic pneumococcal pneumonia; however, more recently, Sligl and Hoang et al found that macrolides did not improve 30 day mortality in critically ill patients with pneumonia [32]. On the other hand, Giamarellos- Bourboulis et al [33] and Walky et al [34] reported that use of macrolide antibiotics improved survival and clinical outcomes and in patients with acute lung injury and suspected gram negative infections. The mechanism of this effect likely reflects beneficial dampening of the host inflammatory response to the bacterial products. The limitation of our study was lack of bacterial burden data at various points during in vivo experiment. We collected mice blood once only at the end of experiment (after 5 days of GBS infection or earlier if mice died). These data at different points during experiment would be highly informative, however, it would have confounded our clinical score due to hypovolemia and shock symptoms secondary to frequent blood draw and circulatory volume loss in mice.

Many studies over the last 20 years have also demonstrated that significant improvements in clinical symptoms and survival have been reported in patients with diffuse panbronchiolitis, cystic fibrosis and bronchiolitis obliterans syndrome, following the addition of macrolides to therapy [35]. The clinical efficacy of macrolides in these diseases is not directly related to their antimicrobial activity [36]. In our experiments, we found that the lower mortality and morbidity of mice treated with combination antibiotics persisted even while using azithromycin resistant GBS. These findings suggest that the beneficial effects of azithromycin are mediated by reducing inflammation in GBS infected mice. Hiwatashi et al [37] showed that azithromycin suppresses mitogen- or superantigen-induced proliferation of PBMCs by possibly inhibiting both cellular JNK and ERK activities. P. Cornacchione et al [38] have shown that GBS survives in macrophages and their intracellular survival may be secondary to impairment of PKC signal transduction. It is possible that the protective effect of AZM in our study may either be related to effect of AZM at the level of toll like receptor, MyD88 or downstream signaling molecules of inflammatory cascade.

We found that addition of azithromycin to ampicillin resulted in decreased cytokine responses to azithromycin-susceptible or azithromycin-resistant GBS isolates *in vitro* and *in vivo*. Most importantly, addition of azithromycin to ampicillin therapy of mice with GBS sepsis caused by either azithromycin-susceptible or azithromycin-resistant strains resulted in less severe disease (lower clinical sepsis scores) and markedly improved survival. To our knowledge, this is the first study examining the use of macrolides for GBS sepsis in murine model. Our study adds to growing evidence that antibiotic combinations that dampen the inflammatory response may improve outcomes in serious infections. Our data challenge the traditional management of GBS sepsis and potentially point us towards better adjuvant therapies of these infections. Future studies understanding the mechanisms of these findings may be helpful in paving way to clinical trials of management of sepsis.

## Materials & methods

### Reagents & antibiotics

Dulbecco's modified Eagle's medium (DMEM) was purchased from Mediatech Inc. (Herndon, VA). L -glutamine was purchased from Invitrogen (Carlsbad, CA). Fetal bovine serum was purchased from Hyclone Laboratories (Logan, UT). The following antibiotics were purchased from the Department of Pharmacy at Le Bonheur Children's Hospital: 1) Ampicillin for injection, USP (Apothecon B.V., Barneveld, The Netherlands); 2) Azithromycin for injection; 3) Sterile water for injection, USP (American Pharmaceutical Partners, Inc.) was used for both the reconstitution and dilution of antibiotics to the desired concentrations. For *in vitro* experiments clinically achievable concentrations of antibiotics were used as follows: ampicillin 20 μg/mL, azithromycin 5μg/mL and 20μg/mL. These antibiotic concentrations were equal to or exceeded the minimum inhibitory concentration (MIC) of the GBS isolate by at least 10-fold. For *in vivo* studies, the dose of ampicillin was 100 mg/kg/dose q24 hours and azithromycin dose was 10mg/kg/dose or 50mg/kg/dose q24h. All antibiotics were administered intraperitoneally.

### Infectious agents

A previously studied [39] type-Ia GBS isolated from a neonatal patient with sepsis was used. Serotyping was performed by the Centers for Disease Control and Prevention (Atlanta, GA). The GBS Ia isolate was susceptible to antimicrobials used in this study with MIC as follows: ampicillin ≤0.25 mcg/L, azithromycin <0.125mcg/L. MIC was determined by the clinical microbiology laboratory at Le Bonheur Children's Hospital using the VITEK 2 system (bio Mérieux, Marcy l'Etoile, France). This isolate was maintained in serial culture from glycerol stock maintained at -80°C. GBS type Ia R (azithromycin resistant) strain was obtained from neonatal clinical isolate from Le Bonheur Children’s Hospital in Memphis TN, which was preserved in microbiology department. The MIC of resistant azithromycin strain was tested with E-test (MIC = 16 mg/L). The stock isolates were maintained at -80^o^ C. Bacteria were routinely sub-cultured in TSB (trypticase soy broth [BD, Franklin Lakes, NY]) with 5% CO2 at 37°C, and were used after washing three times with PBS. Final concentrations of 10^6^ and 10^7^ cfu/mL were used *in vitro* experiments and of 10^7^ and 10^8^ for *in vivo* experiments, based upon our previous experience [21] and our preliminary studies.

### Cells and cell culture

RAW 264.7 cells were purchased from America Type Culture Collection (Manassas, VA) and cultured in antibiotic-free Dulbecco's modified Eagle's medium (DMEM) supplemented with 10% fetal bovine serum and 2 mM l-glutamine.

### *In vitro* experiments

Experiments were done in 24-well tissue culture plates with 1 × 10^6^ cells/well. Macrophages were stimulated for 18h with GBS Ia or azithromycin resistant GBS 10^6^ or 10^7^cfu/ml. In all GBS experiments, ampicillin (20 mg/L) alone or ampicillin + azithromycin (5 mg/L or 20 mg/L) was added to the cell culture immediately before the bacteria. After 18 h stimulation, cell supernatants were collected for cytokine (TNF- α) determination by ELISA (eBioscience, SanDiego, CA). In addition, experiments were performed using RAW 264.7 murine macrophages stimulation with antibiotics without GBS (negative control) and with GBS type 1a without any antibiotics (positive control). Furthermore, RAW 264.7 cells were also stimulated with heat killed GBS (positive control). Cell viability assay with trypan blue exclusion method was performed for experimental conditions with RAW cells and GBS.

Mice Three week old Swiss Webster mice were obtained from The Charles River Laboratory. All animal care and housing requirements set forth by the National Institutes of Health Committee on Care and Use of Laboratory Animals of the Institute of Laboratory Animal Resources were followed, and animal protocols were reviewed and approved by the University of Tennessee Animal Care and Use Committee.

### *In vivo* experimental design

Dose response experiments were performed initially to develop an effective GBS sepsis mouse model with adequate GBS inoculum. Swiss Webster female mice, 18g of mean bodyweight, were injected intraperitoneally with 10^5^–10^9^ cfu/ml GBS type Ia to 2 mice each. These mice were observed every 24 hours for signs of sepsis using a clinical sepsis score. This scoring tool was developed using data from body conditioning score for mice [40], which is used for rapid assessment of health status in mice. Body conditioning score correlates with mice health status better compared to other data such as weight or WBC counts [40]. In addition, data regarding physical activity, unkempt hairy coat and timing of onset of moribund status or death were noted. Each of these 4 variables were scored from 0 to 2, where 0 was considered normal to mild change and 2 was considered most severe status. Cumulative scores were calculated in each group at the end of each 24 hours of GBS infection. The clinical sepsis score was validated for assessing severity of clinical symptoms after GBS infection.

After initial dose response experiments, GBS inocula of 10^7^ and 10^8^ cfu/ml were considered optimal because these mice developed symptoms within 48 hours. One ml GBS inoculum (10^7^ cfu/ml) was administered intraperitoneally in all mice during initial experiment. Similar experiments were also done using GBS inoculum 10^8^ cfu/ml. Subsequently, we divided mice into 4 different groups as follows.

Group I: GBS infected mice and no antibiotics: phosphate buffer solution (PBS) = control group.

Group II: GBS infected mice treated with ampicillin

Group III: GBS infected mice treated with azithromycin

Group IV: GBS infected mice treated with combination of ampicillin+azithromycin

The first dose of antibiotic or PBS was given after 12 hours of intraperitoneal GBS injection. Antibiotics administered once every 24 hours, intraperitoneal, and doses were 100 mg/kg/dose for ampicillin and 10 or 50 mg/kg/dose for azithromycin. Mice were observed (blinded observer) every 12 hours and were sacrificed after 5 days of GBS injection or earlier if they reached a moribund state. Blood samples were obtained by cardiac puncture at the time of death. Blood culture for bacterial clearance and serum TNF- α, IL-1, IL-6, IL-10 and MIP-1α levels by ELISA technique (eBioscience, SanDiego, CA) were measured.

### Statistical analysis

The mean and standard deviation of cytokines and chemokines in each group were calculated. Cytokine levels for *in vitro* (TNF-α by RAW 264.7 cells) and *in vivo* (serum IL-6 and TNF-α) experiments were compared between groups using analysis of variance (ANOVA). After confirming these groups were different, post hoc analysis was performed between ampicillin vs azithromycin and ampicillin vs AMP+AZM group. Similar analysis was performed to compare mean clinical scores in GBS infected mice treated with different antibiotics at each time point 24 hours after GBS injection. The p value ≤0.01 was considered significant.
